# A Unique Case of Relapsing Polychondritis Presenting with Acute Pericarditis

**DOI:** 10.1155/2013/287592

**Published:** 2013-12-17

**Authors:** John V. Higgins, Uma Thanarajasingam, Thomas G. Osborn

**Affiliations:** ^1^Department of Medicine, Mayo Clinic, 200 1st Street SW, Rochester, MN 55905, USA; ^2^Division of Rheumatology, Department of Medicine, Mayo Clinic, Rochester, MN 55905, USA

## Abstract

Relapsing polychondritis (RP) is an inflammatory disease of the cartilaginous tissue primarily affecting the cartilaginous structures of the ear, nose, joints, and the respiratory system. Cardiovascular complications of RP are associated with high morbidity and mortality and occur most commonly as valvular disease. Pericarditis is a less common complication, occurring in 4% of patients with RP and has not previously been described at presentation. We describe a case of relapsing polychondritis with acute pericarditis at presentation.

## 1. Introduction

Relapsing polychondritis (RP) is an autoimmune disorder of unknown etiology primarily affecting the cartilaginous structures of the body. It may also involve other noncartilaginous, proteoglycan rich organs [1]. It is a relatively rare disease with no animal model, making investigation into the mechanism of disease more difficult. Due to this, RP is often difficult to diagnose and treat, with life threatening consequences, if proper diagnosis is not made [2]. The disease is episodic and progressive, with a heterogenous phenotype [3]. The most common presenting features include auricular chondritis, seronegative arthritis, nasal chondritis, ocular inflammation, and laryngotracheal symptoms [1, 3].

Infection and respiratory problems are the most common cause of death, but cardiac complications are the next most common cause of mortality [4]. Valvular involvement is the most common cardiac cause of both morbidity and mortality. Other less common cardiac complications include conduction disturbances, pericarditis, vasculitis, and vascular disease such as aortic aneurysm or dissection [5]. Cardiovascular complications almost universally present later in the disease course with a mean interval of six years after presentation [5]. Only AV nodal conduction abnormalities, presenting as a third degree heart block, have been reported in the literature at presentation of RP [6]. Acute pericarditis is a relatively rare complication of RP, present in only 4% of patients during the course of the disease, and has never been reported at the time of presentation [5]. We present the case of a patient diagnosed with RP who was found to have acute pericarditis on presentation.

## 2. Case Report

A 31-year-old woman presented to the emergency department with chest and facial pain. She had no significant past medical history. She was in her usual state of health until 6 weeks prior to presentation when she developed rhinitis and cough. She had been treated with antibiotics with no relief. She then developed pain and swelling on the bridge of her nose, bilateral cheeks, and bilateral eyelids. She was treated with a brief course of corticosteroids with mild relief in symptoms. She was evaluated by an otolaryngologist 2 weeks before presentation; she was given a diagnosis of facial cellulitis and was restarted on antibiotic therapy.

Four weeks prior to presentation, she developed migratory pleuritic-type chest pain and associated tenderness to palpation over the areas of pain. Her symptoms improved in the upright position. Her chest and facial pain became intolerable, and she presented for further evaluation.

**Figure 1:**
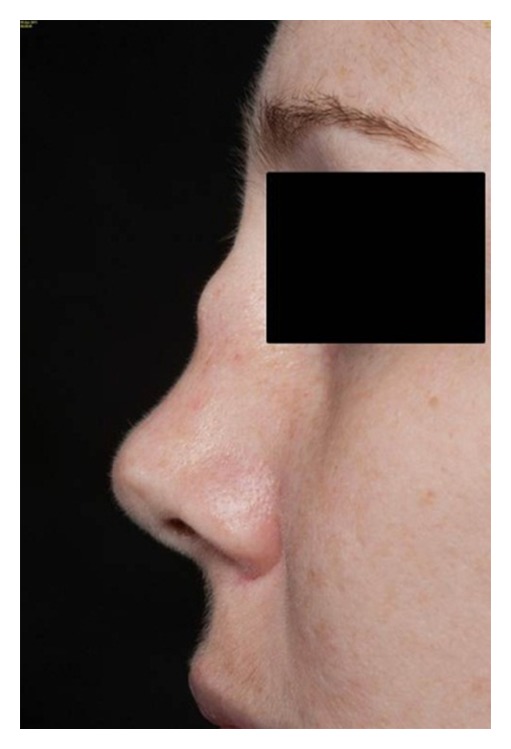
Acute onset saddle nose deformity due to inflammation of the nasal cartilage demonstrated six weeks following symptom onset.

On physical exam, she was noted to have a saddle nose deformity with edema of the nasal mucosa (Figure 1) and pain to palpation over the costal cartilage of the right 4-5th ribs. There was no auricular inflammation, tracheal tenderness, or synovitis. Cardiac exam was normal without cardiac rub. Systems review was negative for nasal crusting or epistaxis, diminished hearing or vision, paresthesias, or any sensory loss.

Initial laboratory data was remarkable for a sedimentation rate of 46 mm/hr (0–29 mm/hr), C-reactive protein of 104.4 mg/L (<8 mg/L), hemoglobin of 10.7 g/dL (12.0–15.5), and a mean corpuscular volume of 88.6 fL (81.6–98.3 fL). Antineutrophil cytoplasmic antibodies (ANCAs), antinuclear antibodies, rheumatoid factor, and creatinine were all unremarkable. Influenza, respiratory syncytial virus, and human immunodeficiency virus studies were negative.

**Figure 2:**
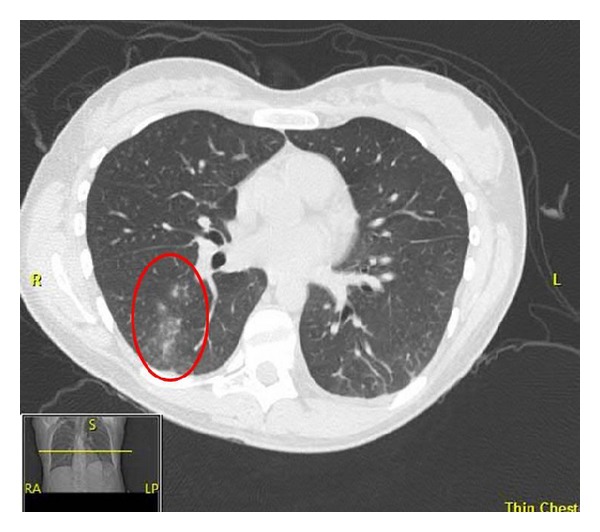
Ground glass opacities in the right lower lobe consistent with an acute inflammatory process.

**Figure 3:**
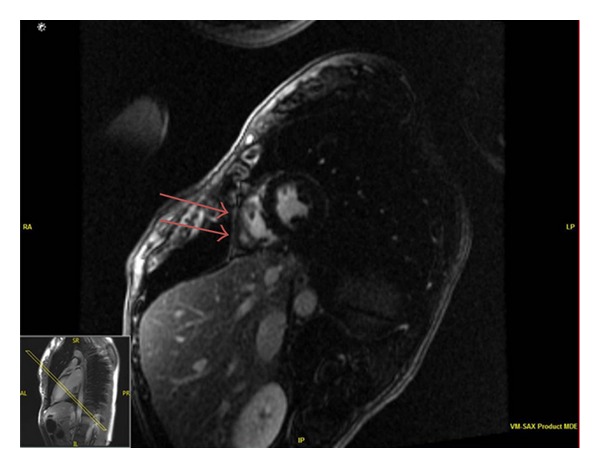
Pericardial enhancement of the right ventricular free wall on cardiac MRI consistent with acute pericarditis.

Computed tomography (CT) scan of the chest was performed with contrast and revealed a nonspecific, ground glass nodular infiltrate of the right lower lobe (Figure 2). A transthoracic echocardiogram (TTE) revealed a pericardial effusion around the right atrium with basal inferior and inferoseptal hypokinesis without valvular disease. A subsequent cardiac MRI showed pericardial enhancement over the right ventricular free wall consistent with acute pericarditis (Figure 3). Ophthalmologic exam was negative for uveitis or other pathology. Given the findings of nasal chondritis, acute noninfectious pericarditis, nonspecific ground glass opacities by CT scan possibly secondary to inflammation, and elevated inflammatory markers, as well as the lack of serologic or clinical findings for ANCA-associated vasculitis, a clinical diagnosis of RP was made.

She was started on prednisone 30 mg daily, colchicine 0.6 mg daily, and dapsone 50 mg daily. She was then dismissed from the hospital with improvement in her symptoms.

Approximately 8 weeks following discharge, while tapering prednisone to 20 mg daily, she had recurrence of facial pain and swelling and developed auricular inflammation. Bilateral nasal biopsies were performed to rule out vasculitis. This was notable for mild to moderate inflammation, with no granulomas or evidence of vasculitis. Her prednisone dose was increased, and symptoms resolved. She continues on dapsone and a prednisone taper with plans to undergo nasal reconstructive surgery in the upcoming year. She has had no recurrence of chest pain.

## 3. Discussion

The diagnosis of relapsing polychondritis is typically clinical; there is no specific serologic test for RP [3]. The McAdams criteria were the initial diagnostic criteria [7] and required three out of six of the following: bilateral auricular chondritis, nonerosive seronegative inflammatory arthritis, nasal chondritis, ocular inflammation, respiratory tract chondritis, and audiovestibular damage. Modified criteria have been proposed by Damiani and Levine [8] which include having 1 McAdam criterion plus tissue diagnosis or 2 McAdam criteria plus response to corticosteroids. The Michet criteria include proven inflammation in 2 of 3 areas—auricular, nasal, or laryngotracheal cartilages—as well as two of the following: ocular inflammation, vestibular dysfunction, seronegative arthritis, and hearing loss [4]. This patient had nasal chondritis, acute pericarditis, and likely respiratory chondritis, as well as a robust response to corticosteroids. Additionally, her relapse of severe inflammation of the nasal cartilage and ear discomfort during the initial prednisone taper supports the diagnosis as well.

Cardiovascular complications are responsible for approximately 18% of deaths in RP patients trailing only pulmonary and infectious causes of death [9]. These can occur at any point in the disease course. With advanced diagnostic techniques, pericarditis may be a more frequently recognized component of RP. It is important to differentiate between RP and other connective tissue diseases as they have variable complications and disease courses. Due to the potential for these life threatening complications, cardiac surveillance with echocardiogram should be performed at all stages of disease, including presentation [9]. Potential serious complications such as atrial flutter have been associated with acute pericarditis in RP [10].

Immunosuppressive therapy is the mainstay of treatment for RP for both active disease and cardiac complications. Prednisone is the traditional mainstay of therapy with nonsteroidal anti-inflammatory drugs and dapsone for milder disease. Cyclosporine A and cyclophosphamide are typically reserved for refractory disease and organ threatening damage [1]. Active flares including cardiac complications have been reported to occur with suspension of therapy [11].

Cardiovascular complications which are not amenable to treatment with medical therapy may require further intervention to treat the underlying problem. Pacemaker implantation is required in patients who develop high grade AV block [9]. Additionally, patients who develop severe valvular regurgitation causing symptomatic heart failure require surgical repair. Aortic valve involvement is more common than mitral valve involvement, with no reported complications of the tricuspid or pulmonary valves [9]. In addition, aortic root dilation may occur, leading to aortic regurgitation in 10% of patients [12]. This may require surgical replacement. When pericarditis has been reported in RP, it has not been associated with hemodynamic compromise, but it should be treated appropriately with nonsteroidal anti-inflammatory drugs and/or colchicine. Pulmonary disease was also evident in our patient with nonspecific inflammation and ground glass opacities shown on CT of the chest. Inflammatory airway edema is the likely inciting factory for lower respiratory tract disease in RP and this precedes the more worrisome respiratory finding of airway stenosis [13].

This case highlights a rare presenting sign of relapsing polychondritis. Cardiac complications should be monitored at all stages of the disease course with appropriate intervention to prevent further, potentially fatal consequences.
